# In vitro activity of hyaluronic acid and human serum on periodontal biofilm and periodontal ligament fibroblasts

**DOI:** 10.1007/s00784-023-05121-z

**Published:** 2023-06-28

**Authors:** Xilei Zhu, Livia von Werdt, Graziano Zappalà, Anton Sculean, Sigrun Eick, Alexandra Stähli

**Affiliations:** Department of Periodontology, School of Dental Medicine, University of Bern, Bern, Switzerland

**Keywords:** Periodontal therapy, Cross-linked hyaluronic acid, Periodontal ligament fibroblasts, Gingival fibroblasts, Interleukin-8, Antibiofilm activity

## Abstract

**Objectives:**

A beneficial effect of cross-linked hyaluronic acid (cHA) on periodontal wound healing and regeneration has recently been demonstrated. The present in vitro study was designed to obtain deeper knowledge on the effect of cHA when applied in the gingival sulcus (serum-rich environment) during non-surgical periodontal therapy.

**Materials and methods:**

The influence of cHA, human serum (HS), and cHA/HS on (i) a 12-species biofilm formation, (ii) the adhesion of periodontal ligament fibroblasts (PDLF) to dentine surface, (iii) the expression and secretion of interleukin-8, and (iv) the expression of receptors of HA in PDLF and gingival fibroblasts (GF) were evaluated.

**Results:**

At 4 h of biofilm formation, cHA and HS in combination (cHA/HS) slightly decreased the colony-forming unit counts in biofilm whereas the metabolic activity of biofilm was reduced in all test groups (cHA, HS, cHA/HS) vs. control. At 24 h, the quantity of biofilm was reduced in all test groups vs. untreated control. The test substances did not affect adhesion of PDLF to dentin. HS increased the expression of IL-8 by PDLF and GF which was partially downregulated by cHA. HS and/or cHA promoted the expression of the HA receptor RHAMM in GF but not in PDLF.

**Conclusions:**

In summary, the present data indicate that serum neither negatively affect the activity of cHA against periodontal biofilm nor had any unwanted influence on the activity of PDLF.

**Clinical relevance:**

These findings lend additional support for the positive effects of cHA on cells involved in periodontal wound healing, thus pointing to its potential use in non-surgical periodontal therapy.

## Introduction

Hyaluronic acid (HA), also named hyaluronan, is a glycosaminoglycan and a major component of the extracellular matrix of vertebrate tissues, abundant in almost all body fluids such as synovial fluid or serum [1]. It is synthesized as a high-molecular weight polymer of 1000–6000 kDa, but can be degraded to low-molecular weight of less or equal 250 kDa or further fragmented to oligos [1]. In medicine, HA has become increasingly important as formulations used in wound healing, the treatment of osteoarthritis or of respiratory and urinary tract infections, and in tissue and regenerative medicine [2]. With respect to the oral cavity, HA is present in saliva [3], gingival crevicular fluid [4], and the soft periodontal tissues [5].

Periodontitis, a disease leading to the destruction of the tooth-supporting tissues, is characterized by an interaction of a dysbiotic biofilm with host response leading to an ongoing inflammatory state [6–8]. Therapy of periodontitis always includes the removal of the subgingival biofilm [9]. The subgingival area of a teeth is bathed in the gingival crevicular fluid, which corresponds to a serum transudate in periodontal health and a serum exudate in periodontal disease [10]. It contains many factors, e.g., immunoglobulins, antimicrobial peptides, or proteases, being involved in the immune response [11]. Periodontal ligament fibroblasts play a special part in periodontal tissue regeneration in the first place by establishing a new attachment [12]. Moreover, they are central players in innate immunity as in inflammation they produce many mediators including proinflammatory cytokines such as interleukin (IL)-8 [13].

In vitro data have shown that a cross-linked hyaluronic acid (cHA) enhanced the expression of genes encoding type III collagen and transforming growth factor-β3, characteristic of scarless wound healing. Moreover, the cHA upregulated the expression of genes encoding pro-proliferative, pro-migratory, and proinflammatory factors and positively influenced the proliferative, migratory, and wound healing properties of different cell types involved in periodontal wound healing/regeneration [14]. These positive biologic effects of cHA on periodontal ligament cells have recently been confirmed in a series of experimental studies providing histological evidence for periodontal regeneration in intrabony, recession, and furcation defects following regenerative surgery and application of cHA [15–17]. Results from controlled clinical studies have provided further evidence on the potential clinical relevance of using cHA in regenerative periodontal surgery in intrabony and recession defects [18, 19].

Systematic reviews underlined a beneficial effect of HA on clinical outcomes (periodontal probing depth (PPD) reduction, less bleeding on probing (BOP), clinical attachment level (CAL) gain) of surgical and non-surgical periodontal therapy [20, 21]. In the included studies, different formulations of high-molecular weight HA and of different origins were applied [21]. In a recent RCT, a gel formulation was used which contained mainly cross-linked high-molecular weight HA added by a small amount of natural high-molecular weight HA [22]. After 3 months of non-surgical periodontal therapy, differences in BOP and PPD reduction were clearly in favor of the HA-treated group [22]. Using adjunctively the gel formulation in residual pockets resulted in by trend (not statistically significant) better results vs. instrumentation alone after 12 months [23].

The aim of this in vitro study was to get deeper knowledge of the effect of cHA when applied in the gingival sulcus during non-surgical periodontal therapy. The focus was on the interaction of microorganisms and periodontal fibroblasts against the background that serum is an essential component of gingival crevicular fluid. We analyzed the influence of cHA on (i) biofilm formation, (ii) the adhesion of periodontal ligament fibroblasts to tooth surface, (iii) the expression and secretion of interleukin-8, and (iv) the expression of receptors of HA in periodontal fibroblasts.

## Materials and methods

### HA and human serum preparation

As hyaluronic acid formulation a commercially available product (Hyadent BG®, Regedent AG, Zurich, Switzerland) was used. According to the manufacturer’s information, the product (cHA) contains 16 mg cross-linked HA (molecular weight of about 1000 kDa) and 2 mg natural HA per ml.

Human serum (HS) was purchased from Sigma-Aldrich (Merk KGaA, Darmstadt, Germany). In the assays, cHA was used in concentrations of 12.5 mg/ml (0.225 mg/ml HA), 25 mg/ml (0.45 mg/ml HA), and 50 mg/ml (0.9 mg/ml HA) and HS in concentrations of 12.5 mg/ml, 25 mg/ml, and 50 mg/ml. When cHA was used in combination with serum (cHA/HS), the respective concentrations each of both were 12.5 mg/ml, 25 mg/ml, and 50 mg/ml. A 0.9% w/v NaCl solution was the negative control.

### Microorganisms

A 12-species periodontal biofilm was used in this study:

1. *Streptococcus gordonii* ATCC 10558
2. *Actinomyces naeslundii* ATCC 12104
3. *Fusobacterium nucleatum* ATCC 25586
4. *Campylobacter rectus* ATCC 33238
5. *Parvimonas micra* ATCC 33270
6. *Eikenella corrodens* ATCC 23834
7. *Treponema denticola* ATCC 35405
8. *Prevotella intermedia* ATCC 25611
9. *Capnocytophaga gingivalis* ATCC 33624
10. *Porphyromonas gingivalis* ATCC 33277
11. *Tannerella forsythia* ATCC 43037
12. *Filifactor alocis* ATCC 33099

All strains (except for *T. denticola* which was maintained in Mycoplasma broth (BD, Franklin Lake, NJ)) were cultured on Schaedler agar plates (Oxoid, Basingstoke, UK) with 5% sheep blood, in an anaerobic incubator or with 5% CO_2_ (*S. gordonii*) at 37 °C. The bacteria were suspended in 0.9% w/v NaCl according to McFarland 4. One part *S. gordonii* was mixed with two parts *A. naeslundii,* and four parts of the other nine species.

### Cell culture

Human gingival fibroblasts (GF) and human periodontal ligament fibroblasts (PDLF) were harvested from freshly extracted and donated teeth from patients who had been informed of the use of their teeth for research purposes and signed written agreement. As these biomaterials were irreversibly anonymized, no additional approval of the Cantonal ethical committee (KEK) was needed according to the respective guidelines.

The procedure was as described recently [24, 25]. GF and PDLF were cultured in DMEM (Invitrogen, Carlsbad, CA, USA) supplemented with 10% fetal bovine serum (FBS; Invitrogen). For experiments, cells were used between the third and fifth passage. Cells from two donors were included.

All cells were incubated with 5% CO_2_ at 37 °C.

### Activity on periodontal biofilm formation

First, wells of 96-well plates were coated with each 10 µl 1.5% bovine serum albumin (BSA, SERVA Electrophoresis GmbH, Heidelberg, Germany) in phosphate-buffered saline (PBS) for 1 h to generate a proteinaceous layer. Then, 10 µl of test substances (cHA, HS, cHA/HS, final concentration each 50 mg/ml) and the control were added for 30 min incubation. Thereafter, microbial suspension mixed with cultivation broth (Wilkins–Chalgren broth, Oxoid, Basingstoke, UK) in a volume ratio of 1:9 was additionally added, i.e., 200 µl per well. Thereafter, the plates were incubated in an anaerobic incubator, 37 °C for 4 h or 24 h.

At 4 h and 24 h, three different aspects of periodontal biofilm formation were measured: (a) colony-forming units (cfus), (b) biofilm mass, and (c) metabolic activity. Then, following a short, careful washing, 100 µl of 0.9% w/v NaCl were added. Biofilms were scraped from the surface and mixed. One aliquot of the suspension was serially diluted, plated on Schaedler agar plates. The cfus were counted after 8 days of anaerobic incubation. Biofilm quantity was measured by using crystal violet staining, and metabolic activity was determined by Alamar blue staining assay as described before [25].

### Activity on adhesion of PDL fibroblasts to dentine specimens

The dentin discs (about 4 × 4 × 1 mm) were prepared as described recently [26]. The teeth were donated from patients who had been informed of the use of their teeth for research purposes and signed written agreement. As these biomaterials were irreversibly anonymized, no additional approval of the Cantonal ethical committee (KEK) was needed according to the respective guidelines. The dentine discs were placed in 24-well plates in the laminar flow. Then the discs of the serum groups (HS, CHA/HS) were coated with 10 µl of serum (undiluted) for 5 min in the laminar flow and thereafter those of the cHA groups (cHA, CHA/HS) with 10 µl of cHA (undiluted for 5 min). The controls were left uncoated.

Detached PDL fibroblasts were suspended in cell culture (with 1% FBS) to a density of 5 × 10^6^/ml. After a short dipping of the test specimens into 0.9% w/v NaCl, each 1 ml of the cell suspension were added per well. The plates have been incubated with 5% CO_2_ for 72 h. Then after short washing and fixing the cells with methanol, the attached cells were counted. The results represent the mean of 10 fields (mm^2^). The statistical unit was the dentine specimen.

### Release and expression of interleukin-8 and HA receptors by gingival and PDL fibroblasts

For determining cytokine level, each well of 48-well plates was covered with 25 µl of the test substances (final concentrations 12.5 mg/ml, 25 mg/ml, and 50 mg/ml each) for 30 min incubation (RT). Afterwards, 225 µl cell suspension was added at a density of 5 × 10^5^ cells/well (GF and PDLF). After 18 h of incubation (37 °C, 5% CO_2_), the media were collected and centrifuged. From the supernatants, the protein level of IL-8 was quantified by ELISA kits (R&D Systems Europe Ltd., Abingdon, UK) following the manufacturer’s instructions.

For measuring mRNA expression of the cytokine IL-8 and also of the HA receptors (CD44, RHAMM, TLR2, and TLR4), GF and PDLF were seeded into 6-well plates at a density of 5 × 10^5^ cells/well for 18 h. Then, after careful washing, culture medium with 0.5% FBS and the test substances in concentrations of each 25 mg/ml were added for 1 h. After 3 times PBS wash, total RNA was extracted following the instruction of innuPREP RNA Mini Kit 2.0 (Analytic Jena GmbH, Jena, Germany). Then, the GoScript™ Reverse Transcription System (Promega, Madison, WI, USA) was used to reverse 1000 ng RNA into cDNA. Quantitative RT-PCR was carried out by GoTaq® qPCR Master Mix (Promega) with the QuantStudio 3 Real-Time PCR System (Thermo Fischer, Waltham, MA, USA) to determine the mRNA expression level of the cytokine IL-8, and HA receptor genes (CD44, RHAMM). The primer sets are given in Table 1. Gene expression was normalized by GAPDH and analyzed by the 2^−△△CT^ method.

**Table 1:** Primer sequences used for qRT-PCR

Gene	Forward/reverse primers	Primer sequences 5’-3’	References
IL-8	F	GAG AGT GAT TGA GAG GTG GAC CAC	[27]
	R	CAC AAC CCT CTG CAC CCA GTT T	
CD44	F	GAC CTC TGC AAG GCT TTC AAT A	# M59040.1
	R	CAA AGG CAT TGG GCA GGT CT	
RHAMM	F	AGG ACC AGT ATC CTT TCA GAA ATC	# BC017793.1
	R	AGT GCA GCA TTT AGC CTT GC	
GAPDH	F	GAC AGT CAG CCG CAT CTT CT	[28]
	R	TTA AAA GCA GCC CTG GTG AC	

### Statistical analysis

All experiments were performed in at least two independent experiments in each quadruplicate (eight independent biological samples). Log_10_ transformation was used in the case of cfu counts.

Statistical analysis was performed with Kruskal–Wallis test and followed by Mann–Whitney *U* test (with Bonferroni correction) using SPSS 26.0 (IBM Corporation, New York, NY, USA). For qRT-PCR results, one-way ANOVA and Dunnett’s multiple comparisons test were carried out by Graphpad Prism 9 (Graphpad Software, California, USA). Statistical significance was set at *p* < 0.05.

## Results

### Periodontal biofilm formation

In median, the untreated biofilm consisted of 6.59 log10 cfu at 4 h and of 8.90 log10 cfu at 24 h.

**Fig. 1:**
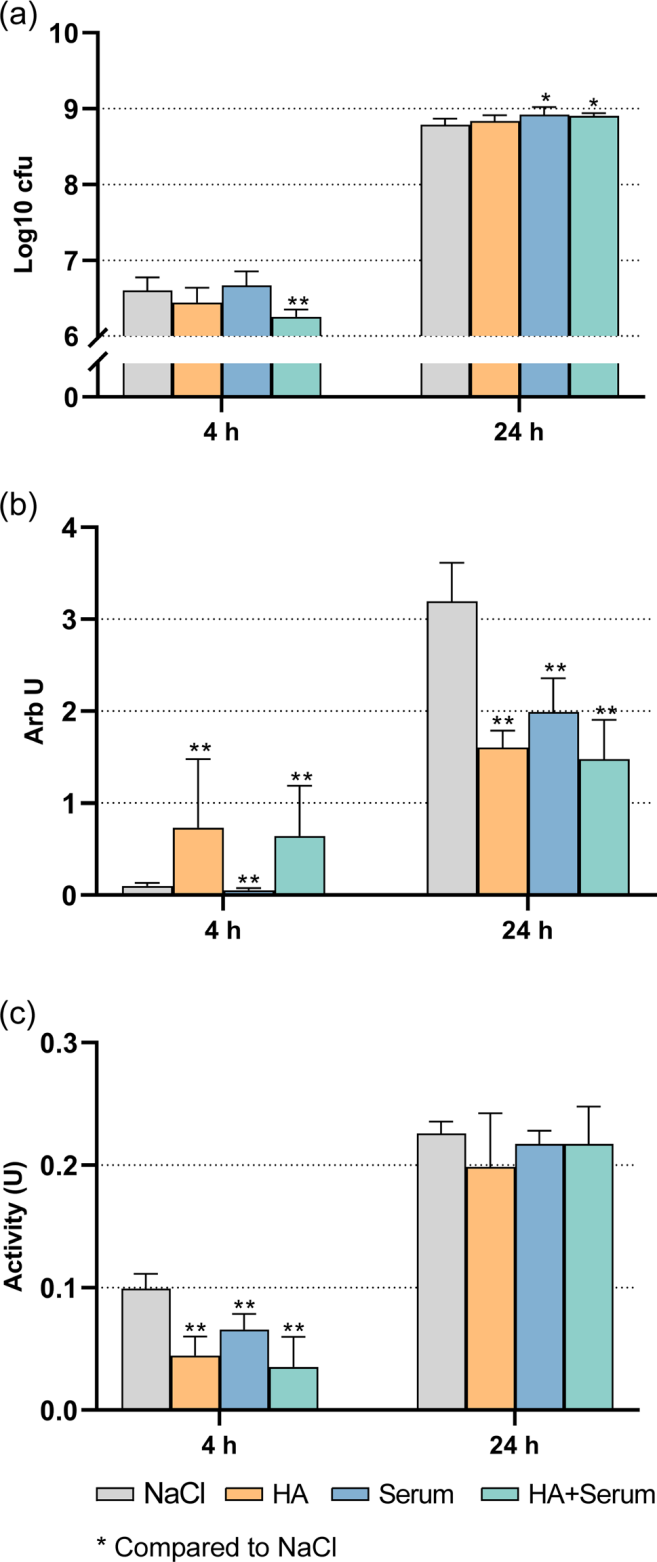
Influence of coating with 50 mg/ml hyaluronic acid (cHA), human serum (HS), and each 50 mg/ml hyaluronic acid/human serum (cHA/HS) on periodontal biofilm formation after 4 h and 24 h. **a** Colony-forming units (cfu); **b** quantity; **c** metabolic activity. Median incl. 25 and 75 percentiles; *p < 0.05, **p < 0.01 vs. control

When applying test substances, there were only minor differences in the cfu counts. The highest difference vs. control was − 0.36 log10, when 50 mg/ml cHA/HS were applied at 4 h (*p* < 0.001; Fig. 1a). At 24 h, all test substances increased the cfu counts; however, differences were in median 0.10 log10 (cHA, *p* = 0.483) to 0.13 log10 (HS, *p* = 0.015).

In terms of biofilm quantity (Fig. 1b), at 4 h, both cHA and cHA/HS groups had higher values compared to the control group (each *p* < 0.001). The quantity of biofilm in HS group decreased (*p* = 0.009). However, at 24 h, the quantity of biofilm was reduced in all test groups vs. untreated control (*p* < 0.001 each).

In all test groups, the metabolic activity of the biofilm was reduced compared to the control at 4 h (*p* < 0.001 each). At 24 h, no difference was found anymore (Fig. 1c).

### HA and attachment of PDL fibroblasts to dentin

**Fig. 2:**
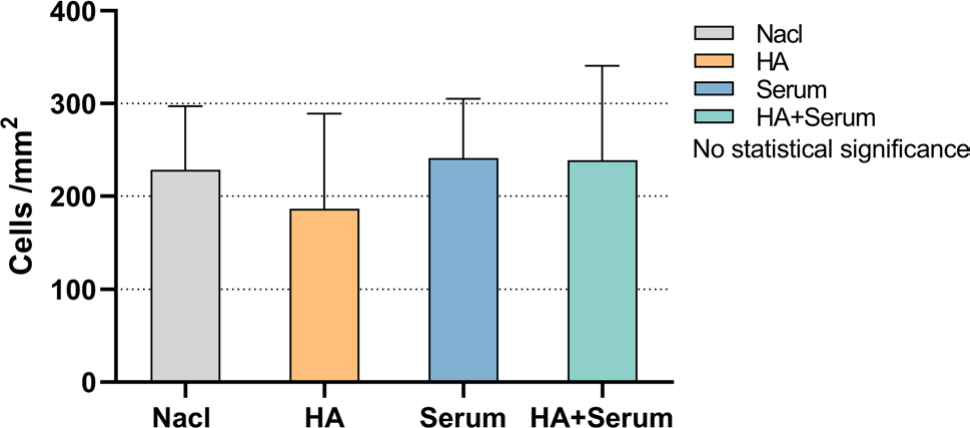
Influence of coating dentine surfaces with hyaluronic acid (cHA), human serum (HS), and hyaluronic acid/human serum (cHA/HS) on attachment of periodontal ligament fibroblasts. Mean and SD

An important step in the resolution of periodontal tissue destruction is a promoted adhesion of fibroblasts to tooth surfaces in the periodontal pocket. Here, the influence of HS and cHA on the number of adhered PDL fibroblasts was studied. There was a minor trend (not statistically significant) to a reduced attachment when the surface was coated with cHA. In case of coating with HS and cHA/HS, the numbers remained unchanged (Fig. 2).

### HA receptors expression in oral fibroblasts

Two crucial HA receptor genes were checked in gingival and PDL fibroblasts. Each 25 mg/ml of test substances (HS, cHA) were used for the mRNA expression experiments.

**Fig. 3:**
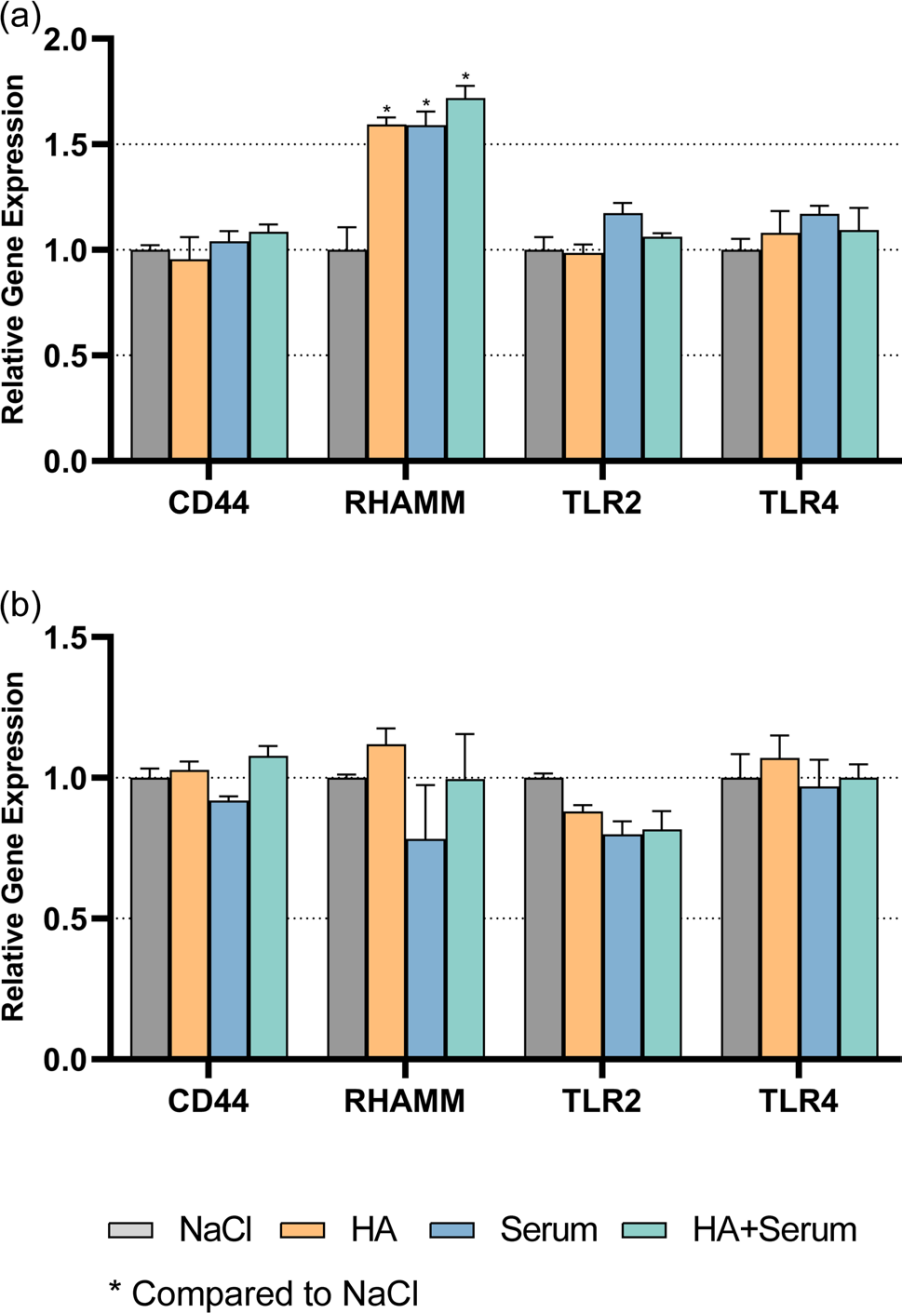
Influence of 25 mg/ml hyaluronic acid (cHA), human serum (HS), and each 25 mg/ml hyaluronic acid/human serum (cHA/HS) on mRNA expression of hyaluronic acid receptors (CD44, RHAMM) in **a** gingival fibroblasts (GF), and **b** periodontal ligament fibroblasts (PDLF). Mean ± SD, *p < 0.05, **p < 0.01 vs. control

The analyzed receptors (CD44, RHAMM) were expressed by both fibroblast types. An influence by the test substances was minor despite reaching in part statistical significance. In gingival fibroblasts (Fig. 3a), all test groups (cHA, HS, cHA/HS) increased the mRNA expression of RHAMM (*p* = 0.003, *p* = 0.003, *p* = 0.001). In PDL fibroblasts (3b), the receptors’ mRNA expression did not statistically significantly differ among the groups.

### Interleukin-8 expression in oral fibroblasts

Both mRNA expression and protein expression of interleukin-8 (IL-8) were measured in GF and PDLF, in which 25 mg/ml cHA and HS were used for mRNA expression whilst 5, 25, and 50 mg/ml were used for protein expression.

**Fig. 4:**
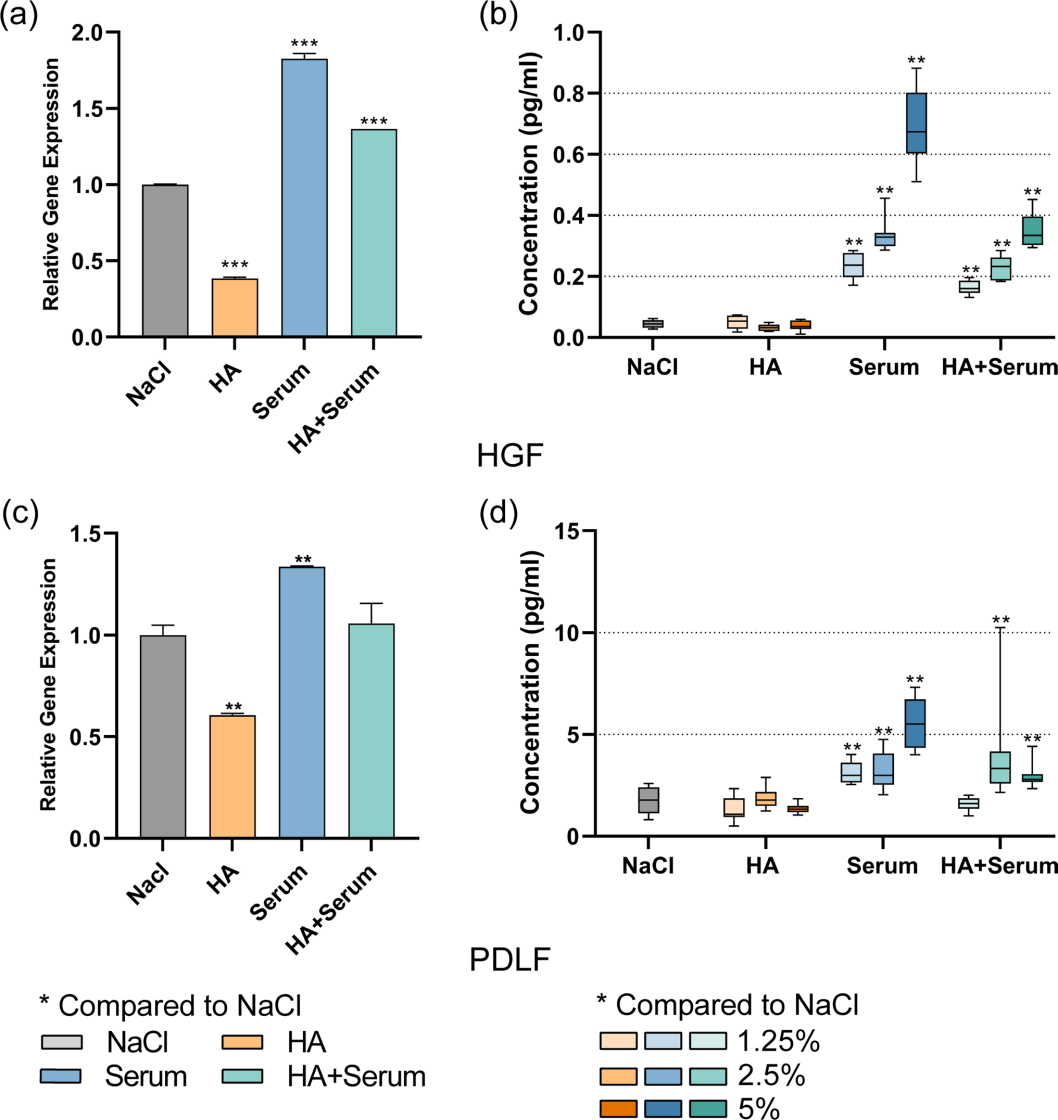
Influence of hyaluronic acid (cHA), human serum (HS), and hyaluronic acid/human serum (cHA/HS) on mRNA expression (**a**, **c**) and protein level (**b**, **d**) of interleukin-8 in **a**, **b** gingival fibroblasts, and **c**, **d** periodontal ligament fibroblasts. mRNA expression: mean ± SD, protein: median incl. 25 and 75 percentiles. *p < 0.05, **p < 0.01 vs. control

As shown in Fig. 4a and c, cHA decreased the mRNA expression of IL-8 in the two fibroblast types (GF: *p* < 0.001, PDLF: *p* = 0.005). At protein level, there was no statistically significant difference for any of the tested cHA concentrations vs. control neither with GF nor PDLF. In contrast, HS significantly increased IL-8 expression in GF and PDLF at the mRNA level (*p* = 0.001, *p* = 0.009). At protein level, results were accordingly, after each tested concentration of cHA higher IL-8 levels were measured vs. non-stimulated GF and PDLF cells (each *p* < 0.001). Also, when cHA was combined with serum, the released levels of IL-8 from GF and PDLF were always higher than from the control (each *p* < 0.001). When comparing the high levels of IL-8 after HS stimulation with those after cHA/HS, the combination with cHA decreased the mRNA expression (GF: *p* < 0.001, PDLF: *p* = 0.002) and also the protein expression (GF all concentrations *p* < 0.001, PDLF 12.5 mg/ml HS vs. 12.5 mg cHA/HS *p* < 0.001, 50 mg/ml HS vs. 50 mg cHA/HS *p* = 0.001).

## Discussion

The present in vitro study has analyzed the effects of a commercial HA product on a periodontal biofilm and periodontal fibroblasts. A product topically used in non-surgical periodontal therapy should inhibit biofilm formation, and, at the same time, positively affect the healing/regenerative potential of the host cells. Gingival crevicular fluid does contain not only serum, but also, besides the presence of serum proteins, a number of other markers involved in the innate and acquired immune response [29].

The results of the present study have shown that both cHA and HS interfered with initial biofilm formation; however, they did not affect adhesion of PDLF to dentin. A further finding was that HS increased the expression of IL-8 by periodontal fibroblasts which was partially downregulated by cHA.

In biofilm formation, bacterial counts were only minorly affected. The strongest effect occurred at 4 h when the surface was coated both with cHA and HS. Interestingly, the results on biofilm quantity were more remarkable. Initially, cHA increased the biofilm quantity since it was probably incorporated in the matrix of the multi-species biofilm. However, at 24 h, the quantity was reduced when the surface was coated with cHA and/or HS. Recently, it was reported that HS inhibited biofilm formation of pathogens including *Staphylococcus aureus*, *Staphylococcus epidermidis*, and *Pseudomonas aeruginosa* although it did not affect planktonic growth, but the addition of an antibiofilm compound could reverse this effect [30]. In the present study, an increased inhibitory effect of cHA on HS was found. Interactions of HS albumin with HA can enlarge the binding ability of HA, as some positive divalent cations, e.g., Ca^2+^, increase the affinity between them and contribute to lubrication [31]. Reported results on HA influence on biofilm formation are controversial. When adding HA on single-species biofilms of respiratory pathogens continuously less biofilm was quantified, the finding was discussed as a disaggregation of the matrix [32]. Also linking a polymethacrylate surface with HA reduced the adhesion of *Staphylococcus epidermidis* [33]. But using HA synthesized by *Streptococcus equi* promoted biofilm formation of *Streptococcus pneumonia* [34]. Regarding bacteria being associated with periodontal disease, recently a decrease by 60% (0.4 log10) of viable counts of *P. gingivalis* after 72 h of biofilm formation was mentioned [35]. An interesting approach seems to be to supplement HA gels with antimicrobials, for example, a HA formulation releasing oxygen reduced *P. gingivalis* growth [36].

In the present study, no clear effect of cHA or/and HS on fibroblast adhesion to dentin surfaces was found. This finding is in line with the results of a recent study [37] where cHA did not change the numbers of adhered PDLF to dentin surfaces. However, it has to be kept in mind that the analysis was made after 8 h and not after 72 h as in our study. Studies on non-cross-linked HA (ncHA) showed an inhibition of fibroblast adhesion and proliferation [33, 38]. cHA is less water soluble and promotes more cell proliferation than ncHA [39]. However, PDLF cultured on plastic surface showed an increase of fibroblast counts (proliferation) by about 20–30% by high-molecular weight HA, irrespective of whether cross-linked or not [40]. Both HA (cHA and ncHA) formulations have a high biocompatibility; in several studies, no negative effect on fibroblast viability was found [14, 40].

As the periodontal fibroblasts also function as immune cells [13], the IL-8 expression was analyzed. IL-8 is one of the most abundant proinflammatory cytokines in the oral cavity; in periodontal disease, it is produced by fibroblasts, epithelial cells, keratinocytes, and macrophages in response to the inflammatory reaction caused by bacteria and their components [41]. HS increased the expression of IL-8 which might be confirmatory to other studies. Serum amyloid A induces the expression of IL-8 in human gingival fibroblasts [42]. HS and its component serum albumin increased the expression of IL-8 by epithelial cells, also after challenging *P. gingivalis* and its obvious ability to degrade IL-8 [43]. IL-8 is a chemoattractant for neutrophils to the site of infection [44]. A positive role of neutrophils in battling the non-balanced microbiota can be assumed; however, neutrophils are also associated with tissue damage [45]. A downregulation of mRNA expression by cHA was found in that study; cHA decreased but did not block IL-8 expression induced by serum. This finding may support a beneficial role in the resolution of inflammation in periodontal therapy. Chen et al. showed that gingival fibroblasts after pretreatment with high-molecular weight HA and thereafter with *P. gingivalis* released less IL-8 in comparison with HA of lower molecular weight [46]. In the inflammatory model of interstitial cystitis, HA showed potent inhibition of IL-8 release [47]. IL-8 binds to HA, the binding is depending on the sulfation degree and the presence of metallic ions [48].

HA is triggering via the receptors RHAMM, CD44, and the intracellular adhesion molecule (ICAM)-1 [2]. Following injury, there is an increased expression of hyaluronic acid receptor genes in the initial stage of inflammation which promotes fibroblasts migration [49]. The focus on the present study was on RHAMM and CD44. The receptors are expressed both by the PDLF and the GF. Expression of CD44 was not affected neither by HS nor by cHA. CD44 is involved in wound healing thereby decreasing inflammatory response [50]. In case of RHAMM, the two types of fibroblasts responded differently to the stimuli. PDLF RHAMM expression did not significantly differ, whereas HS and/or cHA promoted the RHAMM expression in gingival fibroblasts. RHAMM expression is known to be stimulated by low-molecular weight HA. Signaling via the receptor leads to wound closure and resolution of inflammation [51]. The observed increase of RHAMM expression by HS may, at least partly, be responsible for cHA exerting its activity.

Animal models and in vitro research shed light to the role of HA in periodontal regeneration. A study on two wall intrabony defects in dogs which were treated with cHA and a collagen matrix highlighted the role of cHA in promoting periodontal wound healing/regeneration [15]. In diabetic rats, adding cHA to a collagen membrane prevented its premature degradation [52]. In vitro both cHA and ncHA increased early osteogenic differentiation of primary PDL fibroblasts [40]. Both preparations induced proliferation and migration of the fibroblasts and upregulation of genes involved in wound healing and regeneration [14]. In palatal but not in gingival fibroblasts, expression of matrix-metalloproteinases was induced, a finding of relevance when applying palatal transplants in periodontal surgery [14]. Further, the proliferation of mesenchymal stromal and osteogenic progenitor cells was increased by cHA and ncHA [53].

In summary, the present study analyzed the role of cHA in the serum-rich environment of a periodontal pocket. It was shown that the serum did not negatively affect the activity of cHA against periodontal biofilm and on periodontal fibroblasts which, in turn, may support the application of cHA in non-surgical periodontal therapy. However, the present study has also some limitations. First of all, this is an in vitro study which did not consider the complexity of the periodontal region with a plethora of cells interacting with each other. Although here not tested, we assume that similar results concerning the effect of serum and cHA can be expected also for epithelial cells and alveolar bone cells. Second, an interaction of the periodontal biofilm with host cells (e.g., fibroblasts and monocytic cells) was not studied, and third, only one HA formulation (i.e., cHA) was used. Nevertheless, the results of the study encourage further in vitro research including other cell types and interactions with a periodontal biofilm and other HA formulations.
